# TorsinA and the TorsinA-Interacting Protein Printor Have No Impact on Endoplasmic Reticulum Stress or Protein Trafficking in Yeast

**DOI:** 10.1371/journal.pone.0022744

**Published:** 2011-07-26

**Authors:** Julie S. Valastyan, Susan Lindquist

**Affiliations:** 1 Whitehead Institute for Biomedical Research, Cambridge, Massachusetts, United States of America; 2 Department of Biology, Massachusetts Institute of Technology, Cambridge, Massachusetts, United States of America; 3 Howard Hughes Medical Institute, Massachusetts Institute of Technology, Cambridge, Massachusetts, United States of America; Hertie Institute for Clinical Brain Research and German Center for Neurodegenerative Diseases, Germany

## Abstract

Early-onset torsion dystonia is a severe, life-long disease that leads to loss of motor control and involuntary muscle contractions. While the molecular etiology of the disease is not fully understood, a mutation in an AAA+ ATPase, torsinA, has been linked to disease onset. Previous work on torsinA has shown that it localizes to the endoplasmic reticulum, where there is evidence that it plays roles in protein trafficking, and potentially also protein folding. Given the high level of evolutionary conservation among proteins involved in these processes, the ability of human such proteins to function effectively in yeast, as well as the previous successes achieved in examining other proteins involved in complex human diseases in yeast, we hypothesized that *Saccharomyces cerevisiae* might represent a useful model system for studying torsinA function and the effects of its mutants. Since torsinA is proposed to function in protein homeostasis, we tested cells for their ability to respond to various stressors, using a fluorescent reporter to measure the unfolded protein response, as well as their rate of protein secretion. TorsinA did not impact these processes, even after co-expression of its recently identified interacting partner, printor. In light of these findings, we propose that yeast may lack an additional cofactor necessary for torsinA function or proteins required for essential post-translational modifications of torsinA. Alternatively, torsinA may not function in endoplasmic reticulum protein homeostasis. The strains and assays we describe may provide useful tools for identifying and investigating these possibilities and are freely available.

## Introduction

A glutamic acid deletion (ΔE) in torsinA is associated with early-onset torsion dystonia, a devastating autosomal dominant neurofunctional disease that impacts patients as children or young adults and has no cure [1]. The torsinA protein carries a signal sequence in its N-terminus that localizes the protein to the contiguous lumen of the endoplasmic reticulum (ER) and nuclear membrane [2], [3], [4]. C-terminal to this is a hydrophobic region that allows torsinA to interact with membranes [5]. The remainder of the protein encompasses an AAA+ ATPase domain, which includes the site of the dystonia-associated mutation [6].

While AAA+ ATPase domains have been ascribed diverse functions, many are involved in protein remodeling [7]. Yeast have AAA+ ATPases that participate in protein folding, such as HSP104 [8], but none are homologs of torsinA. However, there is precedent for successfully studying AAA+ ATPases in heterologous environments, as HSP104 has been shown to function in neurons, which lack an HSP104 homolog [9].

TorsinA acts in protein trafficking and the dystonia-associated mutation in torsinA disrupts this process [10], [11]. Due to the homology between torsinA and the Hsp100 family, it was hypothesized that torsinA may function as a chaperone. Indeed, recent work has shown that torsinA can untangle protein aggregates *in vitro* and rescue both nematodes and mouse embryonic fibroblasts from ER stress [12], [13]. However, work in PC6.3 cells showed that while the ER stressor dithiothreitol (DTT) induces post-translational modification of torsinA in rat PC6.3 cells, torsinA cannot rescue ER stress or protein aggregation of polyglutamine in these cells [14]. This discrepancy may arise from differences between cell lines. The latter paper was the only one to use a neuronal cell line, which may be the most representative of the cell type impacted by dystonia. TorsinA is present in Lewy bodies (LB), the proteinaceous aggregates that are a hallmark of Parkinson's disease (PD) [15], [16]. TorsinA is able to reduce the aggregation of a principal component of LB, alpha-synuclein (α-syn), in H4 neuroglioma cells, and lessen the neuronal death caused by α-syn expression in nematodes [17], [18]. This aspect of torsinA biology is not understood, since torsinA is localized to the ER [3] and LB and α-syn exist in the cytoplasm [16], [17]. Hence, while data suggest certain cellular functions for torsinA, the details of this role in protein homeostasis is far from understood and it is unclear how the disease-associated mutation of torsinA interferes with its proposed chaperone function in the ER.

Many studies have revealed the utility of modeling complex biology in the yeast *Saccharomyces cerevisiae*. Yeast have an unrivaled genetic toolbox for both hypothesis-based and unbiased high-throughput analyses [19]. Furthermore, yeast have been vital in understanding processes involved in protein homeostasis, including the unfolded protein response (UPR) and the role of chaperones [20], [21]. More recently, yeast have been utilized to model the cell biology of neurodegenerative diseases, including polyglutamine (polyQ) diseases (e.g., Huntington's disease) [22], synucleinopathies (e.g., PD) [23], and Alzheimer's disease [24], [25], [26].

Studies of this nature concerning the cell-biological role of α-syn have been particularly successful. α-Syn elicits toxicity when expressed in yeast, as well as cellular phenotypes reminiscent of those observed in PD patients [23]. From high-throughput screens, our lab and others began to elucidate how α-syn expression alters normal cellular functions. One such screen assayed the consequences of overexpressing individual yeast genes on α-syn-induced toxicity; this revealed conserved cellular processes perturbed by α-syn expression in yeast and higher eukaryotes, including ER-to-Golgi trafficking, and established a link between α-syn and *PARK9*, a gene previously associated with PD by genetic analyses, but not known to be functionally tied to α-syn [27], [28], [29]. A second yeast-based screen discovered chemical compounds capable of rescuing not only α-syn-induced toxicity in neurons, but also toxicity triggered by the drug rotenone, which represents an independent model for PD [30].

Motivated by the success of these prior yeast models, here we attempt to investigate the cellular effects of torsinA expression in *Saccharomyces cerevisiae*. Upon expression of torsinA in yeast, we find that torsinA does not detectably impact protein folding and secretion under the many conditions that we have tested. Although we would not normally report negative results, we do so for several reasons. First, due to the past success of modeling complex diseases in yeast, we foresee others attempting similar studies and hope that this work will serve as a starting point for their analyses. Secondly, it is possible that our failure to detect significant changes upon torsinA expression may be due to missing cofactors, improper protein processing, or subcellular environmental differences between yeast and higher eukaryotes. As these topics are understood in greater detail, this model may come to serve as a useful tool for the discovery of torsinA function and torsinA-interacting partners. For these reasons, all plasmids created in this study will be made freely available from Addgene.

## Results

### Expression of torsinA in the yeast ER requires a yeast ER localization signal

**Figure 1:**
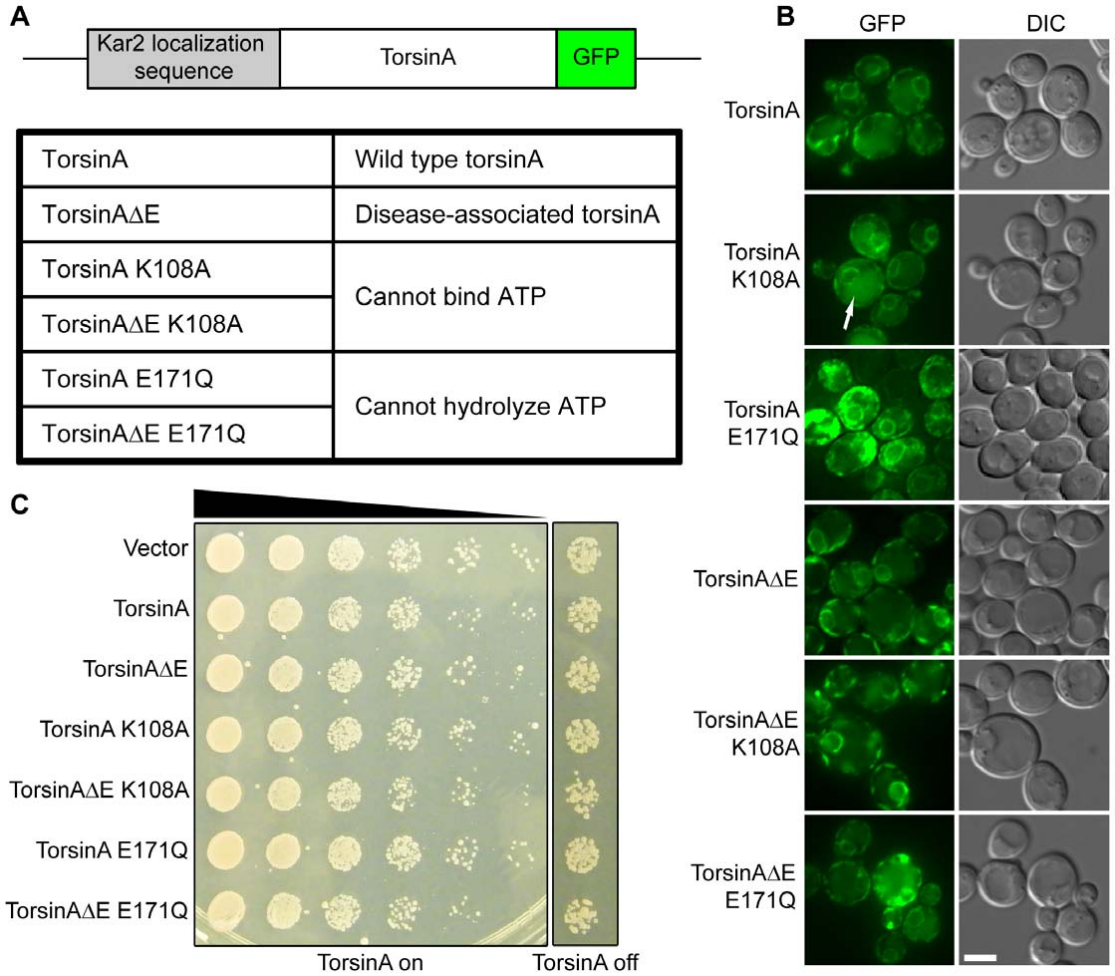
TorsinA can be expressed in the endoplasmic reticulum of yeast. (A) Diagram of constructs created for this work (upper panel) and summary of the different forms of torsinA used in these experiments (lower panel). TorsinA was localized to the endoplasmic reticulum (ER) using the signal sequence of the endogenous yeast protein KAR2 and an HDEL sequence. (B) Microscopy of yeast strains used. TorsinA was localized to the contiguous lumen of the nuclear envelope and ER. Some signal was also seen in the vacuole (arrow head), suggesting a portion of the protein was degraded. A representative frame is shown for each strain. Scale bar  =  2 µM. (C) Growth of torsinA-expressing yeast on plates. Each row is a 5-fold dilution of the previous row. Expression of wild type (WT) or mutant torsinA did not impact the growth rate. Uninduced plates included 1 mM methionine and induced plates lacked methionine.

TorsinA is a resident ER protein [2], [3]. However, expression of full-length torsinA in yeast, driven by a high-expression galactose-inducible promoter, resulted in the formation of large cytoplasmic aggregates (data not shown). Since we were interested in studying the ability of torsinA to act specifically in the ER, we wished to localize the protein to this cellular compartment. To accomplish this, we replaced the endogenous ER localization sequence of torsinA [4] with the localization sequence of the yeast ER-resident protein, Kar2p (Figure 1A) [31]. Importantly, in this construct, only the portion of torsinA that has been shown previously to be cleaved following ER localization was replaced [3], [5], leaving all functional domains of the final protein intact. This caused torsinA to localize to the ER, as visualized by C-terminal green fluorescent protein (GFP) tagging of torsinA (Figure 1B). Even in the ER, the protein still aggregated significantly when expressed from the galactose-inducible promoter (data not shown). Therefore, the expression level of torsinA was decreased by using the MET25 promoter. For all experiments other than microscopy, the GFP tag was replaced with an HDEL (ER retention) motif to ensure that any torsinA mistakenly trafficked to the Golgi apparatus would be returned to the ER [32].

While the data presented below exclusively utilize this form of torsinA, in which the endogenous localization sequence was replaced with that from KAR2, pilot studies were performed using two other fusion constructs. The first attached the KAR2 localization sequence to full-length torsinA and the second utilized torsinA lacking both the endogenous signal sequence and hydrophobic domain. Both of these forms of the protein elicited similar results to those outlined below (data not shown).

Multiple isoforms of torsinA were used in these analyses (Figure 1A). TorsinAΔE, the mutation associated with early-onset torsion dystonia, lacks a glutamic acid at position 302 or 303 [6]. It was hypothesized that this form of the protein may lose torsinA-associated activity, as it has previously been shown that torsinAΔE acts by a dominant-negative mechanism [10]. Furthermore, the ATPase activity of torsinA is required for its chaperone function [13]. Therefore, control constructs were created by inserting two previously characterized mutations in the AAA+ domain into both torsinA and torsinAΔE - K108A or E171Q, which inactivate ATP binding and hydrolysis, respectively [33].

KAR2-tagged torsinA displayed predominantly ER-localized, non-aggregated expression, suggesting the signal sequence was sufficient for relocalization (Figure 1B) [34]. Some cytoplasmic foci were still observed and low-level GFP was also seen in the vacuole (arrowhead points to one example), suggesting that some of the protein was being degraded (Figure 1B). We performed a spotting assay to assess the impact of torsinA expression on the yeast growth rate. None of the forms of torsinA examined impaired or accelerated the rate of growth, suggesting that torsinA expression did not significantly perturb any yeast cellular processes essential for growth (Figure 1C). Importantly, torsinAΔE did not diminish the growth rate of yeast, suggesting that the mutation does not act by a dominant, gain of function mechanism to impede cell viability in our model.

### Expression of torsinA in yeast does not allow for recapitulation of its roles in protein homeostasis and trafficking

**Figure 2:**
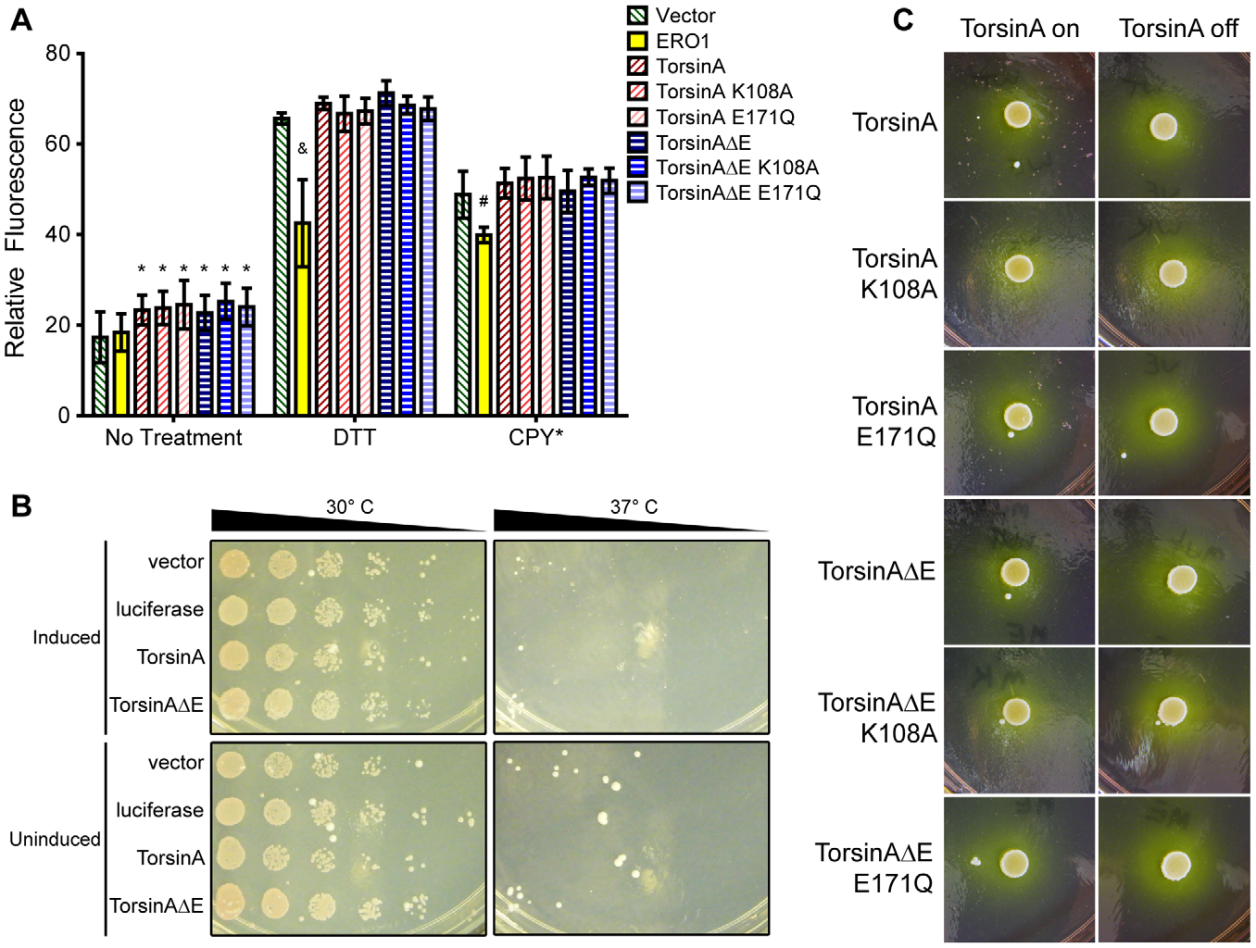
TorsinA does not impact the unfolded protein response or trafficking in yeast. (A) A construct containing GFP driven by a unfolded protein response (UPR) sensitive promoter (UPRE-GFP) was used to monitor levels of the unfolded protein response (UPR) upon stress with 1.5 mM dithiothreitol (DTT) or mutant carboxypeptidase Y (CPY*). ERO1 served as a positive control. TorsinA is not able to reduce UPR levels caused by either stressor. Statistical analysis was conducted in comparison to the vector control strain with a 1-tailed Student's t-test. * = p<0.05, # = p<0.005, & = p<0.001. N = 6 independent trials per sample. (B)Growth of ero1-1 in the presence and absence of torsinA at 37°C. Each row is a 5-fold dilution of the previous row. TorsinA is not able to rescue the growth defect by the *ero1-1* mutation. Uninduced plates included 1 mM methionine and induced plates lacked methionine. (C) Trafficking of invertase, as monitored by halos produced by growth of torsinA-expressing strains on plates containing bromocresol purple (BCP). TorsinA does not impact the rate of secretion.

To test if torsinA could function as a chaperone, yeast strains were assayed for ability to overcome protein folding perturbations in the presence of wild type (WT) and mutant torsinA. To monitor the onset of the UPR, we utilized a previously described reporter that consists of GFP driven by four copies of the promoter from a UPR-responsive gene (UPRE), thus allowing dose-dependent monitoring of an upregulation in the UPR [35], [36]. Each torsinA construct elicited a slight, yet significant, basal increase in the UPR in yeast, as compared to an empty vector (Figure 2A), likely due to the expression of extra protein molecules in the ER. This was not torsinA-specific, since the reporter gene luciferase, when expressed in this manner, elicited the same response (data not shown). For this reason, we assayed the ability of WT torsinA to modulate the UPR in comparison to mutant forms of the protein, hypothesizing that any biologically meaningful effects of torsinA expression should be attenuated by the disease-associated mutation and/or the mutations that inactivate ATP binding. None of the torsinA isoforms elicited an UPR that was significantly different from the others, suggesting that the rise in UPR was not due to mutant torsinA dysfunction (Figure 2A).

This assay was used to study the ability of torsinA to impinge upon the UPR induced by oxidative protein damage in yeast by treating torsinA-expressing cells with DTT. DTT causes oxidative ER stress by inhibiting disulfide bond formation, thus eliciting the UPR [36]. Recent work has suggested that a *C. elegans* homolog of torsinA, *ooc-5*, can act as a sensor of intracellular oxidation, as it contains a disulfide bond important for ATP/ADP binding [37]. DTT triggered the UPR in our strains; however, none of the torsinA isoforms tested lowered the levels of DTT-induced UPR (Figure 2A). To ensure that our reporter system was sensitive to the functions of proteins known to reduce oxidative protein damage, we expressed the yeast protein, ero1p. This protein is an oxidase that is necessary for oxidative protein folding and can reduce levels of UPR upon stress [36], [38]. Indeed, we observed that it significantly reduced the level of DTT-induced UPR (Figure 2A).

As a second assay to gauge the ability of torsinA to reduce cellular stress associated with oxidative damage in yeast, torsinA was expressed in a strain containing the *ero1-1* mutation, resulting in a temperature-sensitive ero1p that limits growth at 37°C [38]. Neither torsinA nor torsinAΔE impacted growth of *ero1-1* at this restrictive temperature (Figure 2B).

Next, we asked if torsinA could reduce the levels of the UPR induced by sources other than oxidative stress in yeast. A constitutively unfolded mutant form of carboxypeptidase Y (CPY*) is an ER-associated degradation (ERAD) substrate that can elicit the UPR [39]. Coexpression of any form of torsinA with CPY* had no significant effect on the level of the UPR observed (Figure 2A). Additionally, pilot studies were performed to test the ability of torsinA to reduce the UPR elicited by tunicamycin, a glycoprotein synthesis inhibitor; however, no significant differences were observed (data not shown).

Multiple studies have documented that torsinA plays a role in vesicular trafficking [10], [11], [40]. Similar to the chaperone system that oversees protein folding, secretion is a highly conserved pathway with many proteins displaying extensive homology between yeast and higher organisms [41]. Therefore, we investigated whether torsinA expression altered protein trafficking in yeast. To do so, we utilized a previously established assay for secretion that monitors trafficking of the endogenous yeast protein invertase to the plasma membrane, where it cleaves sucrose, resulting in a local pH change [42]. This change can then be monitored using the pH-sensitive dye bromocresol purple (BCP). Upon spotting torsinA-expressing strains onto BCP/sucrose plates, no change was observed in the diameter of the halo that resulted from this pH change, suggesting that secretion was not impacted by any of the torsinA forms (Figure 2C).

**Figure 3:**
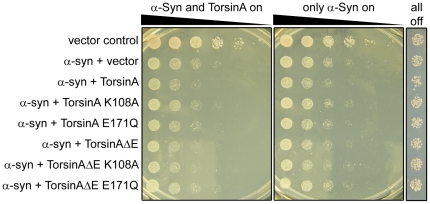
TorsinA cannot rescue α-synuclein-induced toxicity in yeast. Assay showing the ability of yeast to grow in the presence or absence of α-synuclein (α-syn) with and without torsinA. Each row is a 5-fold dilution of the previous row. TorsinA cannot rescue α-syn-induced toxicity.

It has also been suggested that torsinA can prevent the progression of aggregate-associated neurodegeneration in models of synucleinopathies and polyQ disorders [17], [18]. Using the previously described yeast model of α-syn-induced toxicity [23], we asked whether concomitant expression of torsinA and α-syn could rescue this phenotype; however, torsinA had no significant impact on the rate of growth in conjunction with α-syn expression (Figure 3).

### Addition of the torsinA-interacting protein printor is not sufficient to uncover the role of torsinA

**Figure 4:**
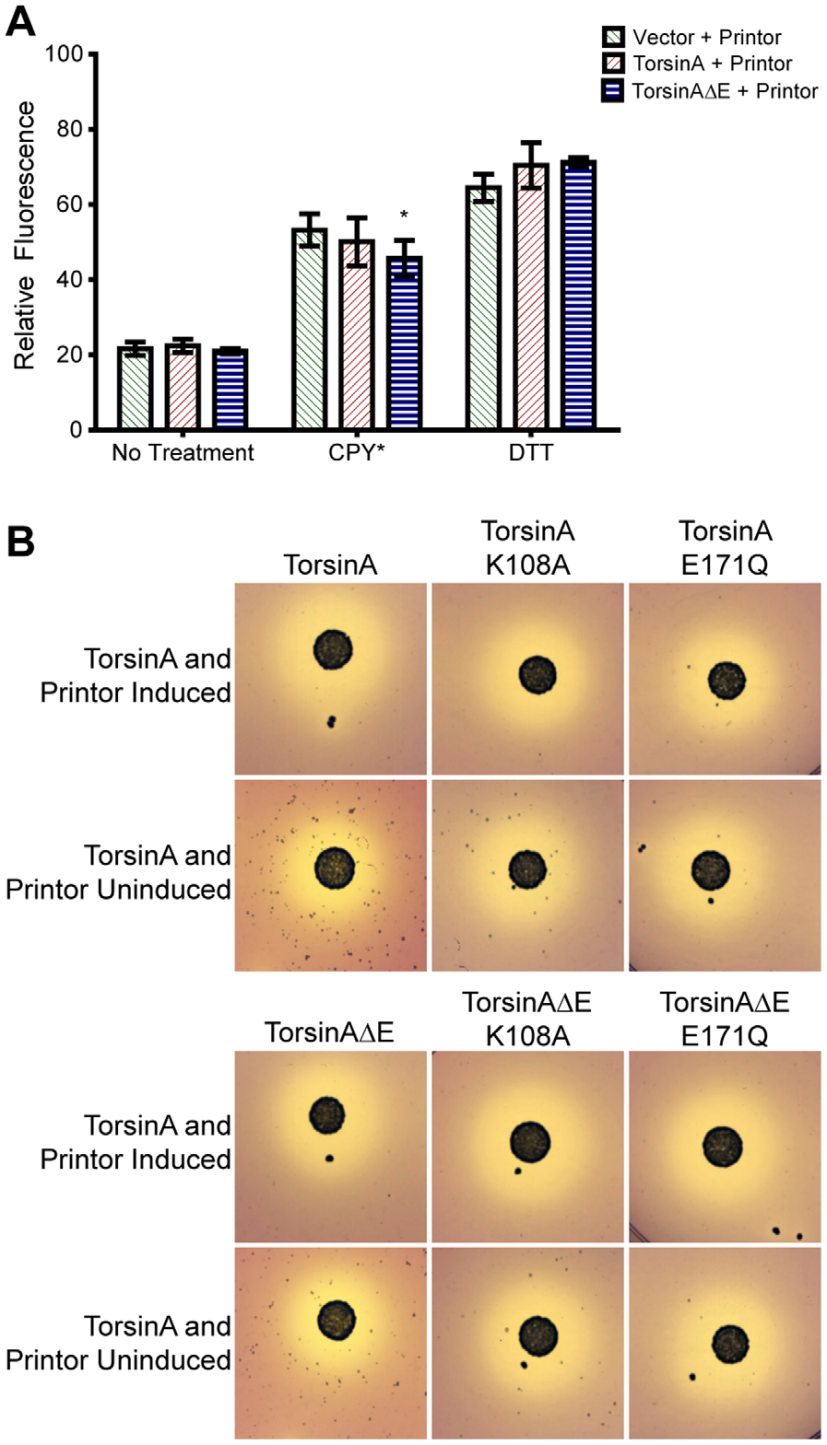
Coexpression of torsinA and printor does not uncover a phenotype stemming from torsinA expression. (A) The UPR of the indicated yeast strains was monitored by flow cytometry to detect expression from the UPRE-GFP construct upon stress with CPY* or 1.5 mM DTT. Coexpression of printor does not allow WT torsinA to reduce UPR-related stress. A 1-tailed Student's t-test was used to compare relative fluorescence of torsinA strains to the vector control. * = p<0.01. N = 6 independent trials per each sample. (B) The impact of torsinA with and without printor on trafficking of invertase. Cells were spotted on plates containing the pH sensitive dye, BCP. Simultaneous expression of torsinA and printor does not impact the rate of secretion.

The above-cited data suggested that torsinA expression in yeast failed to affect any of the cellular processes in which this protein has been shown to act in higher eukaryotic cells. We hypothesized that this might be attributable to the fact that yeast lack certain essential cofactor(s) that are required for torsinA function. Therefore, we reassessed the ability of torsinA to function in the above-stated pathways in the presence of the newly-discovered torsinA-interacting protein, printor [4]. The association between torsinA and printor was discovered through a yeast two-hybrid screen, suggesting that these two proteins could, at a minimum, physically interact within a yeast cell. Furthermore, this prior work demonstrated that printor selectively binds torsinA lacking ATP, suggesting printor could act as a cofactor of torsinA. TorsinA and printor specifically interact in the ER, and not in the nuclear envelope. For this reason, printor was localized to the ER using the Kar2 and HDEL localization sequences, as described above for torsinA. Co-expression of WT torsinA and printor in yeast failed to elicit differential cellular phenotypes upon examination of CPY*- or DTT-induced UPR or invertase trafficking (Figure 4A & B, respectively). TorsinAΔE and printor elicited a small reduction of UPR induction upon co-expression of CPY* (Figure 4A). While this reduction was statistically significant, it is unlikely to be biologically relevant since torsinAΔE was previously show to be inactive.

## Discussion

TorsinA has been proposed to function in two highly conserved cellular processes: protein folding and protein trafficking. As such, we hypothesized that torsinA expression in yeast would impact these pathways. However, we failed to uncover a role for torsinA-mediated control of protein folding and secretion in *S. cerevisiae*. This observation was surprising because other protein folding and remodeling factors, such as HSP90 [43] and HSP104 [9], are readily analyzed when heterologously expressed in evolutionarily divergent organisms. Moreover torsinA was shown to reduce protein aggregation *in vitro*, and we hypothesized that these effects would also extend to our yeast system [12].

However, our present findings can be easily reconciled with this previous work. First, the above-cited *in vitro* study utilized torsinA lacking both its signal sequence and its hydrophobic domain. Secondly, the *in vitro* work, in contrast to previous *in vivo* work in worms with the full-length torsinA protein [13], was unable to uncover a functional difference between torsinA and torsinAΔE [12]. It is possible that the same cofactors or post-translational modifications that are necessary to explain the difference between the *in vitro* and *in vivo* experiments also account for the inability of torsinA to be biologically active in yeast. Alternatively, there may be some difference in the ER environment of the yeast cells as compared to higher eukaryotic cell, which might prohibit its function or be redundant with torsinA activity. Finally, we utilized both a signal sequence and HDEL retention tag to express torsinA in the ER, but previous work [16], [17] suggests it may enter the cytosol, especially in times of proteolytic stress. While the mechanism of this relocalization is not understood, there are many examples of ER proteins being selectively transferred to the cytoplasm when proteostasis be compromised, as occurs wtih ER-associated protein degradation (ERAD) [44]. This would not occur in our model due to the presence of the exogenous HDEL tag. However, when we expressed torsinA directly in the cytosol, with or without α-syn, torsinA had no effect on toxicity (data not shown).

There are a number of other possible explanations for these disparities. While the majority of AAA+ ATPases act as protein remoldeing factors, many have quite specific substrates [7], and it is possible that yeast lack torsinA's substrate(s). Furthermore, the UPR may serve a broader range of functions in vertebrates than in yeast [45] and torsinA may function in an aspect of UPR signaling not conserved in yeast. Finally, as mentioned above, recent evidence suggests that torsinA may not act in general protein homeostasis. Work in neuronal lines has failed to recapitulate the role of torsinA in protein folding, which, noteably, was only seen upon overexpression of torsinA in other models [14]. Furthermore, torsinA has been knocked out in a variety of model organisms, but none of these studies have reported a protein homeostasis-related phenotype. This suggests that torsinA may serve a different principal function, such as its role in maintaining nuclear envelope structure, which has previously been shown to partially explain the neuron-specific sensitivity of torsinA mutation [46].

As more is discovered about torsinA function and its interacting proteins through neuronal culture or transgenic animals, the work presented here may provide a platform for further investigation using yeast. Furthermore, this yeast model might prove helpful in discovering such cofactors, as it could be screened to identify human genes that interact genetically with torsinA. For this reason, all of the plasmids created for this project will be made freely available through Addgene for distribution to others who are interested in testing these hypotheses. While we failed to uncover a phenotype for torsinA expression in yeast, we are optimistic that future work can improve this model and make useful steps toward dissecting the function of torsinA and the dysfunction associated with its mutant form.

## Materials and Methods

### Construction of plasmids

Except where noted, standard Gateway cloning methods were used for cloning [47], [48]. pDONR221 (Invitrogen) was used as the donor plasmid and the pAG series of plasmids [49] were used as destination vectors.

TorsinA and torsinAΔE were obtained as a generous donation from Xandra Breakefield. Overlap extension PCR [50] was used to add the KAR2 and HDEL localization sequences. These constructs were expressed in pAG416Gal-ccdB or pAG416Gal-ccdB-GFP [49]. These were then cut with XbaI and XhoI and subcloned into the same sites of 416-Met25. Site-directed mutagenesis was employed to create the K108A and E171Q mutations using Pfu Turbo (Stratagene).

pRS313-CPY*-HA was a generous gift from Antony Cooper. After sequencing, mutagenic PCR was used to create the same mutation in CPY in pDONR221, which was then used in an LR reaction to move to pAG413-Gal-ccdB [49].

pPW0533, the 304 4xUPRE-GFP reporter, was a generous donation from Peter Walter. Following digestion in within the TRP1 gene, it was transformed into W303 to make a stable strain with an integrated copy.

Printor cDNA was purchased from Kazusa DNA Research Institute (product ID ORK00224) [51]. Overlap extension PCR [50] was used to add the Kar2 and HDEL localization sequences and subclone into pDONR221.

### Primers

Primers used in this work are summarized in Table 1.

**Table 1:** Summary of primers used in this study.

#	Purpose	Sequence
112	Mutagenic PCR TorsinA E171Q	GGTCCATCTTCATATTTGATCAAATGGATAAGATGCATGAGGC
113	Mutagenic PCR TorsinA E171Q	GCCTGCATGCATCTTATCCATTTGATCAAATATGAAGATGGACC
114	Mutagenic PCR TorsinA K108A	GGTGGACAGGCACCGGCGCTAATTTCGTCAGCAAGATCATCG
115	Mutagenic PCR TorsinA K108A	CGATGATCTTGCTGACGAAATTAGCGCCGGTGCCTGTCCACC
121	Overlap extension PCR for KAR2	GGGGACAAGTTTGTACAAAAAAGCAGGCTTCACAAAATGTTTTTCAACAGACTAAGCGC
124	Overlap extension PCR for torsinA	GGGGACCACTTTGTACAAGAAAGCTGGGTTATCATCGTAGTAATAATCTAAC
126	Overlap extension PCR for torsinA	GGGGACCACTTTGTACAAGAAAGCTGGGTTTCACAATTCATCATGATCATCGTAGTAATAATC
138	Overlap extension PCR for torsinA	CCCAGGCTGATGGGCTCCACACCTCTAACTAAAACATTGG
139	Overlap extension PCR for torsinA	CCAATGTTTTAGTTAGAGGTGTGGAGCCCATCAGCCTGGG
140	Mutagenic PCR CPY*	CAAGATTTCCACATCGCTAGGGAATCCTACGCCGGCC
141	Mutagenic PCR CPY*	GGCCGGCGTAGGATTCCCTAGCGATGTGGAAATCTTG
181	Overlap extension PCR for KLHL14	CCAATGTTTTAGTTAGAGGTATGTCCAGATCCGGGGACAGGACCTCCACC
182	Overlap extension PCR for KLHL14	GGGTTTTACAATTCATCATGTTTGTTGTATGGTACACAAGAGGGCAG
183	Overlap extension PCR for KLHL14	GGGGACCACTTTGTACAAGAAAGCTGGGTTTTACAATTCATCATGTTTG
184	Overlap extension PCR for KLHL14	GAAAGCTGGGTTTTTGTTGTATGGTACACAAGAGGGCAG
185	Overlap extension PCR for KLHL14	GGGGACCACTTTGTACAAGAAAGCTGGGTTTTTGTTG

### Yeast strains and growth conditions

With the exception of CKY558 (*ero1-1* containing strain; generous gift from Chris Kaiser) [38], all experiments were done in the W303 genetic background. The standard lithium acetate transformation protocol was used for all yeast transformations [52], [53]. Minimal media included 0.67% yeast nitrogen base without amino acids (Fischer Scientific), supplemental amino acids (minus those needed as selectable markers) (MP Biomedicals), and 2% sugar, with 2% agar included in plates. All liquid growth was performed at 30°C with aeration. Except where noted, growth on plates was performed at 30°C. For galactose induction (CPY* and α-syn), strains were grown overnight in SRaff and diluted into SGal. For methionine induction (torsinA and printor), cells were grown overnight in 1 mM methionine overnight and diluted into media lacking methionine.

### Materials

Dithiothreitol, tunicamycin, methionine, and bromocresol purple (BCP) were purchased from Sigma.

### Spotting assays

Cells were grown overnight in SRaff plus 1mM methionine. Strains were diluted to OD_600_ = 1.0 and serially diluted five-fold for six total repetitions before spotting on the appropriate agar plates.

### UPRE induction assays

Cells were grown overnight in appropriate media. Strains were diluted to OD_600_ = 0.1 in inducing media. DTT was used at the indicated concentrations in order to induce a UPR. After 6 hours of induction, cells were subjected to flow cytometry (Guava PCA-96, Millipore).

### Microscopy

Cells were grown to log phase in non-inducing media, then moved to inducing media for 5 hours. Live cells were visualized by using a Zeiss Axiovert 200 microscope. Z-stacks were taken and deconvoluted using the nearest neighbor algorithm from the Axiovision software.

### BCP trafficking assays

Cells were grown overnight in SD with 1 mM methionine media. They were diluted to an OD_600_ = 1.0 and spotted onto BCP plates containing (as measured in weight/volume) 0.67% yeast nitrogen base without amino acids (Fischer Scientific), supplemental amino acids (minus those needed as selectable markers) (MP Biomedicals), 2% sugar, 2% agar, 0.0032% BCP, and 0.5× PBS. Plates were grown for 3 days at 30°C.

### Statistical Analysis

All data are shown as mean ± standard deviation. A 1-tailed Student's T –test was performed for all comparative statistical analysis.
